# D1- and D2-type dopamine receptors are immunolocalized in pial and layer I astrocytes in the rat cerebral cortex

**DOI:** 10.3389/fnana.2023.1111008

**Published:** 2023-02-14

**Authors:** Satoko Oda, Hiromasa Funato

**Affiliations:** ^1^Department of Anatomy, Graduate School of Medicine, Toho University, Tokyo, Japan; ^2^International Institute for Integrative Sleep Medicine (IIIS), University of Tsukuba, Tsukuba, Japan

**Keywords:** pial astrocyte, protoplasmic astrocyte, pyramidal cell, dopamine receptor subtype, immunohistochemistry

## Abstract

Pial astrocytes, a cellular component of the cerebral cortex surface structure, are observed in a wide range of mammalian species. Despite being recognized as such, the functional potential of pial astrocytes has long been overlooked. Our previous research demonstrated that pial astrocytes exhibit stronger immunoreactivity for muscarinic acetylcholine receptor M1 than protoplasmic astrocytes, indicating sensitivity to neuromodulators. Here, we examined whether pial astrocytes express receptors for dopamine, another crucial neuromodulator of cortical activity. We investigated the immunolocalization of each dopamine receptor subtype (D1R, D2R, D4R, D5R) in the rat cerebral cortex, and compared the intensity of immunoreactivity between pial astrocytes, protoplasmic astrocytes, and pyramidal cells. Our findings revealed that pial astrocytes and layer I astrocytes exhibit stronger D1R- and D4R-immunoreactivity than D2R and D5R. These immunoreactivities were primarily localized in the somata and thick processes of pial and layer I astrocytes. In contrast, protoplasmic astrocytes located in cortical layers II-VI displayed low or negligible immunoreactivities for dopamine receptors. D4R- and D5R-immunopositivity was distributed throughout pyramidal cells including somata and apical dendrites. These findings suggest that the dopaminergic system may regulate the activity of pial and layer I astrocytes *via* D1R and D4R.

## 1 Introduction

Astrocytes in the cerebrum are generally classified into two basic types. Protoplasmic astrocytes reside in gray matter and fibrous astrocytes in white matter. Additionally, there is another type of astrocyte, which is located on the cortical surface just under the pia mater (Tabata, 2015), and that has been identified as pial astrocytes (García-Marqués and López-Mascaraque, 2013), glia limitans astrocytes (Sofroniew, 2020), surface-associate astrocytes (Howe et al., 2008), interlaminar astrocytes (Colombo et al., 1995; Colombo, 2017), and surface astrocytes (Liu et al., 2013; Oda et al., 2018). In this work, we use “pial astrocytes”. The pial astrocytes provide a protective barrier as the glia limitans (Liu et al., 2013; Rua and McGavern, 2018) and the glial processes of the pial astrocytes are rarely associated with blood vessels unlike protoplasmic astrocytes (Howe et al., 2008). Thus, pial astrocytes and protoplasmic astrocytes have distinct localization and morphological properties.

In the late 19th century, several researchers initially reported a type of characteristic neuroglia cell (i.e., pial astrocyte) that is located on the surface of the cerebral cortex. These cells have fibroblast-like somata and thick and long processes toward the deeper cortical layers, based on the observations of Golgi-stained human brains (Andriezen, 1893; Verkhratsky and Nedergaard, 2018). A developmental experiment using *in utero* electroporation of plasmids for combinatorial expression of fluorescent proteins under the GFAP promoter revealed that pial astrocytes near the pia mater are a homogenous population, and distinct from cortical protoplasmic astrocytes (García-Marqués and López-Mascaraque, 2013). Recently, layer-specific RNA-seq and single-cell RNA-seq analyses have shown astrocyte diversity in terms of molecular characteristics and localization (Zeisel et al., 2015; Lanjakornsiripan et al., 2018; Batiuk et al., 2020). Furthermore, it has been reported that there are two populations of astrocytes in the cerebral cortex: those widely distributed in the cortex and low in GFAP expression corresponds to protoplasmic astrocytes, located in layers II–VI, and those located near the cortical surface and rich in GFAP (Zeisel et al., 2018). The former astrocyte corresponds to protoplasmic astrocytes located in layers II–VI, and the latter astrocyte to pial astrocytes and protoplasmic astrocytes in layer I. However, the functional roles of pial and layer I astrocytes are still unknown. In this manuscript, the term layer I astrocytes or astrocytes in layer I is used to indicate protoplasmic astrocytes in layer I and does not include pial astrocytes.

All mammalian brains, including Marsupials, have pial astrocytes with some differences between species. For example, whereas pial astrocytes of primates have long processes extending into the deeper layers beyond layer I, those of rodents have short processes that do not emerge from layer I (Falcone et al., 2019). Furthermore, in primates, pial astrocytes are lined with the narrowly defined glia limitans, a sheet of the astrocytic end-feet, on the cerebral cortical surface, whereas in rodents, pial astrocytes also constitute glia limitans (Liu et al., 2013; Tabata, 2015; Falcone et al., 2019).

Recently, we showed that pial astrocytes have abundant muscarinic acetylcholine M1 receptor (M1R) in the somata and thick processes toward deeper layers. Additionally, the immunopositivity of M1R in pial astrocytes was higher than that in cortical protoplasmic astrocytes (Oda et al., 2018). These findings suggest that acetylcholine regulates the function of pial astrocytes and that other neuromodulators are also involved in the regulation of pial astrocytes. Given that the dopaminergic system is another important neuromodulator of cortical activity, which is involved in voluntary movement, reward, goal-directed behavior, and many neuropsychiatric diseases such as Parkinson’s disease, schizophrenia, and addiction (Ledonne and Mercuri, 2017; Klein et al., 2019), we examined the immunolocalization of each dopamine receptor in pial astrocytes. Additionally, we compared the intensity of immunostaining among pial astrocytes, layer I astrocytes, protoplasmic astrocytes, and pyramidal cells. Mammals have five subtypes of dopamine receptors, from D1R through D5R. They are G protein-coupled receptors; D1-type receptors (D1R and D5R) are GS-coupled and D2-type receptors (D2R–D4R) are Gi-coupled (Ledonne and Mercuri, 2017; Klein et al., 2019).

## 2 Materials and methods

### 2.1 Animals

Seven male adult Sprague-Dawley rats (9–15 weeks old) weighing from 270 to 410 g were used in this study. The treatment and care of the animals were approved by the Institutional Animal Care and Use Committee of Toho University (approved protocol ID 16-52-286).

### 2.2 Primary antibodies and nuclear labeling

The following primary antibodies were used in this study: anti-D1R, -D2R, -D4R, -D5R, and anti-glutamine synthetase (GS) antibodies (Table 1). GS, also known as glutamate-ammonia ligase, is commonly used as a marker for astrocytes. Immunoreactivity for GS was observed in their somata, processes, perisynaptic buds, and end-feet surrounding vessels (Norenberg and Martinez-Hernandez, 1979; Robinson, 2001). The anti-D1R antibody is a rat monoclonal antibody against the C-terminal domain (97 aa) of human D1R (1:300, Cat# D187, RRID:AB_1840789, Clone 1-1-F11 S.E6, Sigma-Aldrich, St. Louis, MO, USA). The specificity of this anti-D1R antibody was previously confirmed *via* western blot using HEK 293T cells transfected with the FLAG-D1R expression construct (Hazelwood et al., 2008), in which this antibody revealed a single band of 50 kDa, corresponding to the molecular weight of D1R. Furthermore, an immunocytochemical examination using HEK 293 cells expressing each dopamine receptor subtype (D1R–D5R) demonstrated that this antibody selectively immunolabeled cells expressing only D1R (Lee et al., 2004). Additionally, western blot analysis of the membrane fraction of rat striatum yielded a single band of 50 kDa.

**Table 1:** Information on primary antibodies.

Dopamine receptor D1 (D1R)	C-terminal domain (97 aa) of human D1R	Sigma-Aldrich; rat monoclonal; Cat# D187, RRID:AB_1840789 (Clone 1-1-F11 S.E6)	1:300
Dopamine receptor D2 (D2R)	Cytoplasmic loop #3 (28 aa) of human D2R	Millipore; rabbit polyclonal; Cat# AB5084P, RRID:AB_2094980	1:500
Dopamine receptor D4 (D4R)	aa 176–185 of human D4R	Calbiochem; rabbit polyclonal; Cat# 324405, RRID:AB_564550	1:3,000
Dopamine receptor D5 (D5R)	C-terminal domain (aa 455–472) of rat D5R	Santa Cruz biotechnology; goat polyclonal; Cat# (R-18) sc-1441, RRID:AB_673640	1:300
Glutamine synthetase (GS)	GS purified from sheep brain	Millipore; mouse monoclonal; Cat# MAB302, RRID:AB_2110656 (Clone GS-6)	1:500

The anti-D2R antibody is a rabbit polyclonal against a cytoplasmic loop #3 (28 aa) of the human D2R (1:500, Cat# AB5084P, RRID:AB_2094980, Millipore, Billerica, MA, USA). The specificity of this antibody was previously confirmed through a study using HEK 293 cells expressing each dopamine receptor subtype (D1R–D5R), demonstrating that it selectively immunolabeled cells expressing only D2R (Lee et al., 2004). Additionally, western blot analysis of c-Myc epitope-tagged D2R using this antibody revealed a single band of approximately 50 kDa, consistent with the molecular weight of D2R.

The anti-D4R antibody is a rabbit polyclonal antibody against aa 176–185 of the human D4R (1:3,000, Cat# 324405, RRID:AB_564550, Calbiochem, San Diego, CA, USA). The specificity of this antibody was previously verified through western blot analysis using membranes of S/9 cells expressing recombinant human D4R (Ricci et al., 2001), which demonstrated that the anti-D4R antibody specifically recognized a single band of 40–42 kDa, corresponding to the molecular weight of D4R. Furthermore, pre-absorption of the antibody with the corresponding blocking peptides resulted in the disappearance of the labeled band. Additionally, it was revealed that this antibody selectively labeled a 40–42 kDa band in the membrane fraction of the rat frontal cortex, which was completely abolished through pre-incubation of the antibody with the immunogen peptide (Ricci et al., 2002).

The anti-D5R antibody is a goat polyclonal antibody against a C-terminal domain of the rat D5R, comprising amino acids 455–472 (Ricci et al., 2001) (1:300, Cat# (R-18) sc-1441, RRID:AB_673640, Santa Cruz Biotechnology, Santa Cruz, CA, USA). The specificity of this antibody was previously verified through western blot analysis using the membrane fraction of HEK 293 cells expressing recombinant human D5R (Ricci et al., 2001). This antibody exhibited reactivity with a band of approximately 51 kDa, which roughly corresponds to the molecular weight of D5R. Pre-adsorption of the antibody with the corresponding blocking peptides resulted in the disappearance of the labeled bands. This antibody was also tested *via* immunofluorescence analysis on HEK 293T cells transfected with either D1R-myc or D5R-myc (Aira et al., 2016). D5R-immunopositivity was detected only in D5R-myc-expressing cells. The labeled bands of D5R-myc were approximately 53 kDa, which corresponds to the molecular weight of D5R.

The GS antibody is a mouse monoclonal antibody against purified GS from the sheep brain (1:500, Cat# MAB302, RRID:AB_2110656, clone GS-6, Millipore, Billerica, MA, USA). The specificity of this antibody was verified through western blot analysis, in which this antibody labeled a single band of approximately 45 kDa, corresponding to the molecular weight of GS (Nasonkin et al., 2011; Kulijewicz-Nawrot et al., 2013). This antibody immunolabeled astrocytes in the prefrontal area of mice (Kulijewicz-Nawrot et al., 2013) and Müller cells in the mouse retina (Nasonkin et al., 2011).

For nuclear labeling, we used Hoechst 33342 (H21492; Thermo Fisher Scientific, Waltham, MA, USA), which elicits a light-blue fluorescence upon binding to the double helix of DNA and subsequent illumination with UV light. The Hoechst 33342 was mixed with a solution containing secondary antibodies.

### 2.3 Tissue preparation

Under anesthesia induced *via* intraperitoneal administration of a mixture of medetomidine (0.3 mg/kg BW, Domitor; ZENOAQ, Koriyama, Japan), midazolam (4 mg/kg BW, Midazolam; SANDOZ, Tokyo, Japan), and butorphanol (5 mg/kg BW, Bettlefar; Meiji Seika Pharma, Tokyo, Japan), rats were perfused with 100 ml of 0.2% heparinized 0.1 M phosphate buffer (PB; pH 7.4), followed by 1,000 ml of 3% paraformaldehyde in 0.1 M PB through the ascending aorta, as previously reported (Oda et al., 2010, 2018). In our experience, fixation with 3% paraformaldehyde occasionally leads to improved preservation of immunoreactivity for G-protein coupled receptors such as dopamine receptors, with structural preservation comparable to that of 4% paraformaldehyde. Following perfusion, the brains were removed and postfixed for 3 h in 3% paraformaldehyde in 0.1 M PB. Blocks of tissue specimens were immersed in 20% sucrose in 0.1 M PB overnight and then sectioned at a thickness of 60 μm with a freezing microtome. The sections were stored in tissue cryoprotective solution (25% glycerol and 30% ethylene glycol in 0.05 M PB) at −80°C until use.

### 2.4 Immunohistochemistry for light microscopy

The sections were washed three times in phosphate-buffered saline (PBS; pH 7.2) and incubated for 5 days at 4°C on a shaker with a primary antibody in 0.1 M PBS containing 2% normal donkey serum. The sections were then washed in PBS and incubated overnight at 4°C on a shaker with one of the secondary antibodies: biotinylated rabbit anti-rat IgG (BA-4000, Vector Laboratories, Burlingame, CA, USA) for D1R detection; biotinylated goat anti-rabbit IgG (BA-1000, Vector Laboratories) for D2R and D4R detection; biotinylated rabbit anti-goat IgG (BA-5000, Vector Laboratories) for D5R detection, at a dilution of 1:200 in PBS containing 2% normal donkey serum. The sections were then washed in PBS and incubated in an avidin-biotin-peroxidase complex solution (ABC kit; PK-4000; Vector Laboratories) overnight at 4°C on a shaker. No detergent was used in all staining processes because in our experience the use of detergents can decrease the immunoreactivity of G protein-coupled receptors. The sections were then washed and incubated in 3,3’-diaminobenzidine (D8001; Sigma-Aldrich), which was diluted at 0.02% with PB containing 0.002% hydrogen peroxide, for 5–10 min at room temperature for visualization. The sections were mounted on gelatin-coated slides, dehydrated in a graded alcohol series, cleared with xylene, and coverslipped with Malinol (Muto Pure Chemicals, Tokyo, Japan). The sections were examined and photographed under a microscope (BX50; Olympus, Tokyo, Japan) equipped with a digital camera (DP70; Olympus).

### 2.5 Immunofluorescence and confocal imaging

The sections were washed three times in PBS and incubated for 4 days at 4°C on a shaker, in the presence of an anti-GS antibody and one of the anti-dopamine receptor antibodies, in a solution of 0.1 M PBS containing 2% normal donkey serum. Subsequently, the sections were washed in PBS. The sections were then incubated in a mixture of secondary antibodies (Alexa 555-conjugated goat anti-rat IgG (1:400, A21434, Thermo Fisher Scientific, Waltham, MA, USA) for D1R detection, Alexa 555-conjugated donkey anti-rabbit IgG (1:400, A31572, Thermo Fisher Scientific) for D2R and D4R detection, Alexa 555-conjugated donkey anti-goat IgG (1:400, A21432, Thermo Fisher Scientific) for D5R detection, Alexa 488-conjugated donkey anti-mouse IgG (1:400, A21202, Thermo Fisher Scientific) for GS detection, diluted with 0.1 M PBS containing Hoechst 33342 (2 μg/ml; H21492; Thermo Fisher Scientific) for nuclear labeling and 2% normal donkey serum for 4 h at room temperature. The sections were washed in PBS, then briefly washed in distilled water, and mounted on slides with ProLong Diamond (P36970; Thermo Fisher Scientific). The cerebral cortex was observed using an LSM-880 confocal laser scanning microscope (Carl Zeiss, Oberkochen, Germany) equipped with a blue diode laser of 405 nm, an argon laser of 488 nm, and a DPSS laser of 561 nm. Images were obtained using the 63× (N.A., 1.4) and 100× (N.A., 1.4) oil immersion objective lenses, and 20X objective lens (N.A., 0.8). To obtain an image with each objective, the pinhole size was adjusted so that the airy unit of Alexa 555 was 1. The optical slice thicknesses were 0.9, 0.9, and 2.0 μm for 63× , 100× , and 20× lenses, respectively. The brightness and contrast of the images were adjusted using image browser software (ZEN2; Carl Zeiss). Images were exported in TIFF format.

## 3 Results

### 3.1 Distribution of each dopamine receptor in the cerebral cortex

**Figure 1:**
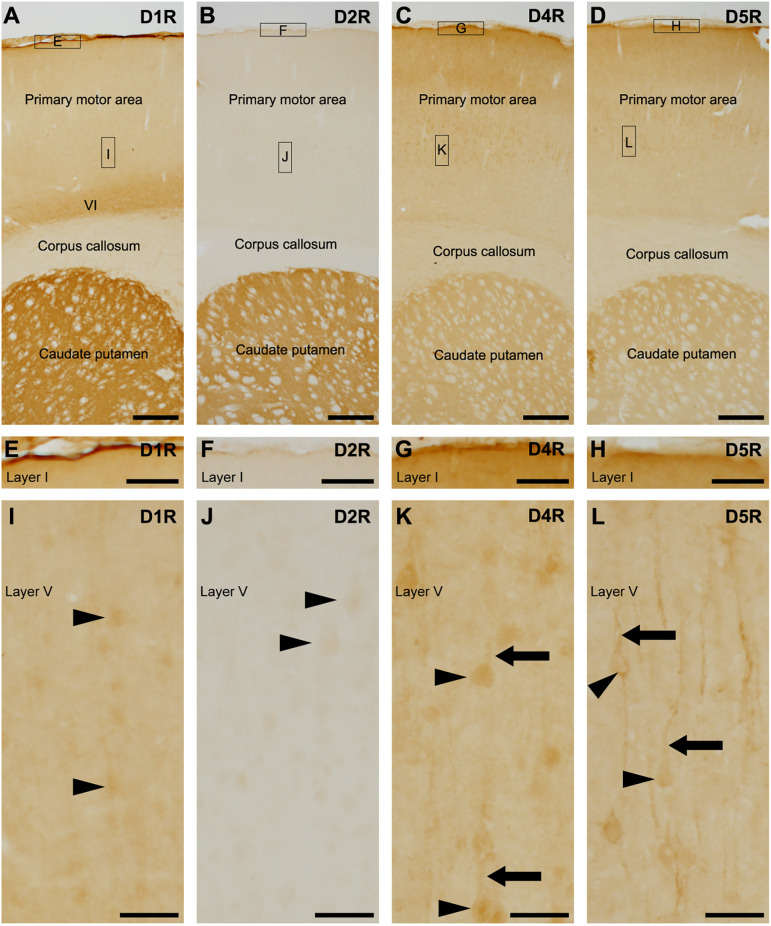
Distribution of each dopamine receptor in the motor cortex and caudate putamen. **(A)** Representative immunohistochemistry of the frontal cortex for D1R. High-magnification images of rectangles indicated with E and I are shown in **(E,I)**. Moderate to strong D1R-immunoreactivities were observed in the pial surface of the cerebral cortex, layer (VI), and caudate putamen. **(B)** Representative immunohistochemistry of the frontal cortex for D2R. High magnification images of rectangles indicated with F and J are shown in **(F)** and **(J)**. **(C)** Representative immunohistochemistry of the frontal cortex for D4R. High magnification images of rectangles indicated with G and K are shown in **(G)** and **(K)**. **(D)** Representative immunohistochemistry of the frontal cortex for D5R. High magnification images of rectangles indicated with H and L are shown in **(H)** and **(L)**. **(E–H)** Immunohistochemical images of the cortical surface for D1R **(E)**, D2R **(F)**, D4R **(G)**, and D5R **(H)**. **(I)** Immunohistochemical images of the cortical layer V for D1R. Neuronal somata show weak immunopositive signals for D1R (arrowheads). **(J)** Immunohistochemical images of the cortical layer V for D2R. Neuronal somata show weak immunoreactivity for D2R (arrowheads). **(K,L)** Immunohistochemical images of the cortical layer V for D4R or D5R. Many neuronal somata (arrowheads) and apical dendrites (arrows) show immunoreactivity for D4R or D5R. Scale bars: 500 μm **(A–D)**; 125 μm **(E–H)**; 50 μm **(I–L)**.

We examined dopamine receptors, D1R, D2R, D4R, and D5R, and exclude D3R because D3R is primarily expressed in the islands of Calleja and the nucleus accumbens, with minimal presence in the cerebral cortex (Diaz et al., 1995; Le Moine and Bloch, 1996; Mrzljak et al., 1996; Mladinov et al., 2010). Pronounced D1R-immunoreactivity was observed on the pial surface of the cerebral cortex and caudate putamen (Figure 1A), with the highest reaction located in layer VI of the cerebral cortex (Figure 1A), which is well in accordance with D1R localization using D1R-tdTomato transgenic mice (Anastasiades et al., 2019). Weak D2R-immunoreactivity was observed in the cerebral cortex, while robust D2R-immunopositivity was found in the caudate putamen (Figure 1B), which is consistent with previous findings using BAC D2-EGFP mice (Khlghatyan et al., 2019). Moderate D4R-immunopositivity was similarly observed in both the cortex and caudate putamen (Figures 1C,D), in accordance with a previous study (Luedtke et al., 1999).

The pial surface of the cerebral cortex exhibited moderate to strong immunoreactivities for D1R, D4R, and D5R, but not for D2R (Figures 1E–H). In layer V of the primary motor area, the neuronal somata-like structure displayed very weak immunoreactivities for D1R and D2R (Figures 1I,J). Weak to moderate immunoreactivities for D4R were observed in neuronal somata and their apical dendrites (Figure 1K), as in a previous report using D4R-EGFP transgenic mice (Noaín et al., 2006). Additionally, moderate immunoreactivities for D5R were observed in the somata and apical dendrites of pyramidal cells (Figure 1L), which is consistent with a previous study (Luedtke et al., 1999).

### 3.2 D1R-immunoreactivities in cortical astrocytes

**Figure 2:**
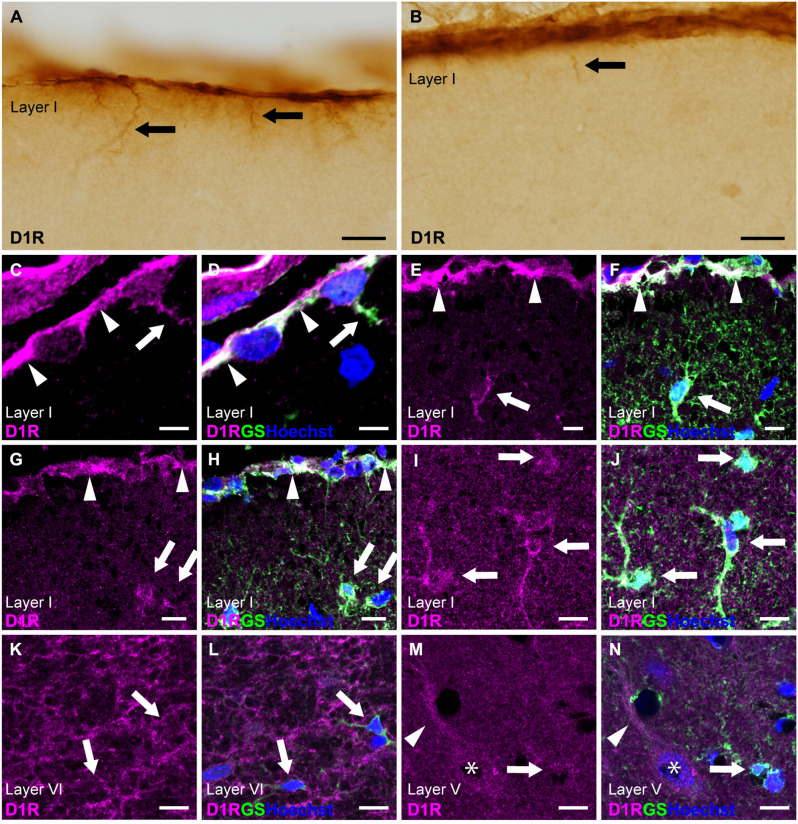
D1R-immunoreactivity in astrocytes. **(A,B)** D1R-immunopositive signals in the cortical surface of the prelimbic **(A)** and somatosensory **(B)** areas. Arrows indicate D1R-immunolabeled processes of pial astrocytes. **(C,D)** Confocal imaging of double immunostaining for D1R (magenta) and GS (green) indicated strong D1R-immunoreactivities of the somata (arrowheads) and process (arrows) of pial astrocytes in the prelimbic area. **(E,F)** Confocal imaging of double immunostaining for D1R (magenta) and GS (green) indicated strong D1R-immunoreactivities of pial astrocytes (arrowheads) and layer I astrocytes (white arrows) in the primary motor area. **(G,H)** Confocal imaging of double immunostaining for D1R (magenta) and GS (green) indicated strong D1R-immunoreactivities of pial astrocytes (arrowheads) and layer I astrocytes (white arrows) in the somatosensory area. **(I,J)** Confocal imaging of double immunostaining for D1R (magenta) and GS (green) indicated strong D1R-immunoreactivities of layer I astrocytes (arrows) in the primary motor area. **(K,L)** In layer VI of the primary motor area, reticular D1R-immunoreactivities were observed. GS-positive protoplasmic astrocytes had weak immunoreactivities for D1R (arrows). **(M,N)** In layer V of the primary motor area, protoplasmic astrocytes (arrows) had weak D1R-immunoreactivity. D1R-immunoreactivities were found in pyramidal cells (asterisks) and their apical dendrites (arrowheads). Nuclei were stained with Hoechst33342. Scale bars: 20 μm **(A,B)**; 5 μm **(C,D)**, 10 μm **(E–N)**.

Next, we examined cortical astrocytes in detail under light microscopy and confocal laser scanning microscopy. We classified cortical astrocytes into three categories: pial astrocytes, layer I astrocytes or astrocytes in layer I, and protoplasmic astrocytes in layers II and deeper. In the prelimbic and somatosensory areas, we observed strong D1R-immunoreactivites restricted to the pial surface of the cerebral cortex and not the adjacent layer I (Figures 2A,B). The cortical surface displayed robust immunopositivity for D1R. D1R-immunoreactive cellular processes were extended from the pial surface into layer I (Figures 2A,B). Double immunostaining for D1R and GS revealed that D1R-immunoreactivity was predominantly present in the somata of GS-positive pial astrocytes (Figures 2C–H), with weak presence in the astrocytic processes extending into layer I. Similarly, astrocytes in layer I displayed moderate immunoreactivity for D1R in their somata and processes (Figures 2I,J). In contrast, GS-positive protoplasmic astrocytes in layers II and deeper exhibited only weak D1R-immunoreactivity (Figures 2K–N). Additionally, we observed moderate immunofluorescence for D1R in layer VI, consistent with light microscopic observation (Figure 1A), which was not overlapped with GS-positive cells (Figures 2K,L). The pyramidal cells of the primary motor area exhibited moderate D1R-immunopositivity in their somata and apical dendrites (Figures 2M,N).

### 3.3 D2R-immunoreactivities in cortical astrocytes

**Figure 3:**
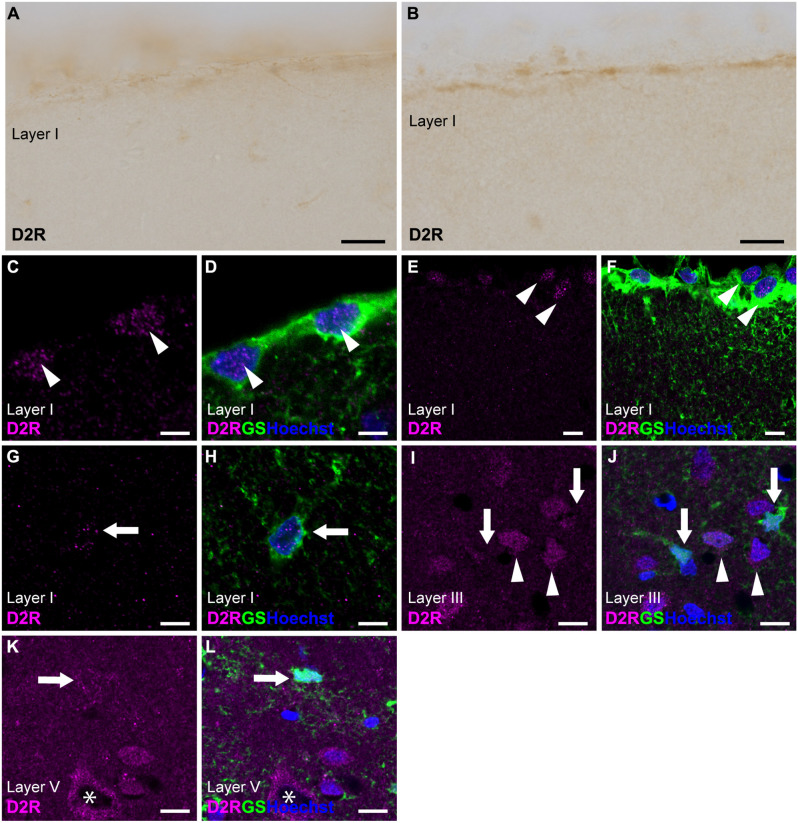
D2R-immunoreactivity in astrocytes. **(A,B)** Very weak immunoreactivities for D2R were observed in the pial surface of the prelimbic **(A)** and somatosensory **(B)** areas. **(C,D)** Confocal imaging of double immunostaining for D2R (magenta) and GS (green) indicated a few weak D2R-immunoreactivities in pial astrocytes (arrowheads) in the prelimbic area. **(E,F)** Confocal imaging of double immunostaining for D2R (magenta) and GS (green) indicated weak D2R-immunoreactivities in pial astrocytes (arrowheads) in the primary motor area. **(G,H)** Weak D2R-immunoreactivities were observed in layer I astrocytes (arrowheads) in the somatosensory area. **(I,J)** In layer III of the primary motor area, D2R-immunoreactivities (magenta) were observed in GS-negative cells (arrowheads), but not in GS-positive protoplasmic astrocytes (arrows). **(K,L)** In layer V of the primary motor area, D2R-immunoreactivities (magenta) were observed in GS-negative cells (asterisk), but only very weak in GS-positive protoplasmic astrocytes (arrows). Nuclei were stained with Hoechst33342. Scale bars: 20 μm **(A,B)**; 5 μm **(C,D)**, 10 μm **(E–L)**.

Immunostaining for D2R revealed the weakest immunoreactivities in the cerebral cortex among dopamine receptors (Figures 1, 3A,B). To elicit immunofluorescent signals for D2R, larger laser power was required than for other dopamine receptors. Double immunostaining for D2R and GS demonstrated that weak D2R-immunoreactivity was almost exclusively detected in nuclear regions of pial and layer I astrocytes (Figures 3C–H). In layers II and deeper, GS-positive protoplasmic astrocytes exhibited low or negligible D2R-immunoreactivity. Several GS-negative cells in layer III showed D2R-immunopositivity (Figures 3I,J). In layer V of the primary motor area, pyramidal cell-like large cells exhibited the immunopositivity for D2R that was confined to the cytoplasmic region of their somata (Figures 3K,L).

### 3.4 D4R-immunoreactivities in cortical astrocytes

**Figure 4:**
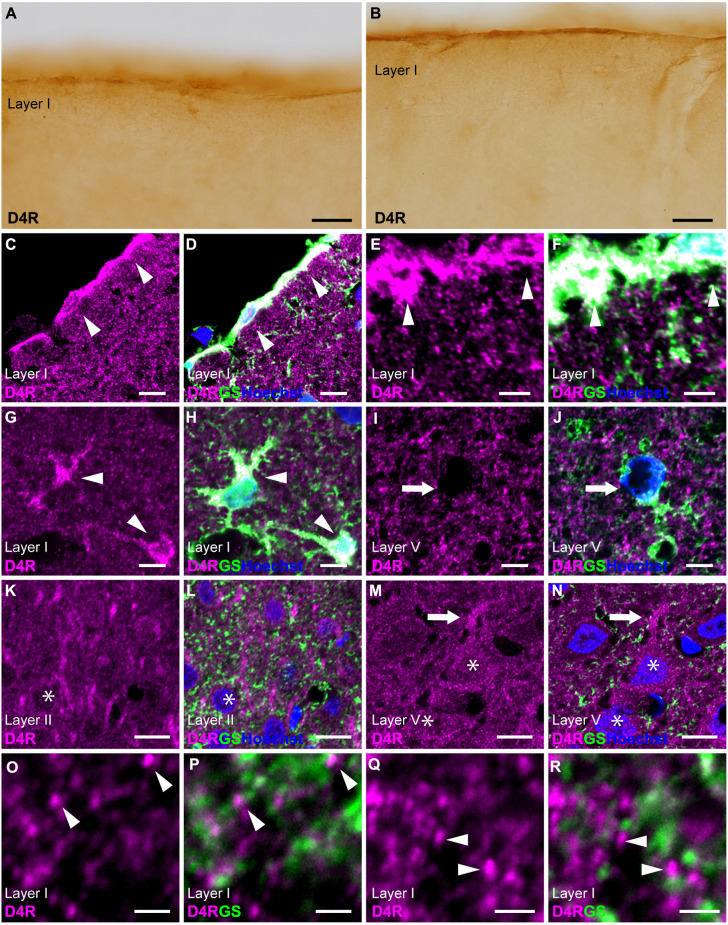
D4R-immunoreactivity in astrocytes. **(A,B)** Moderate D4R-immunoreactivities of the cortical surface of the prelimbic **(A)** and somatosensory **(B)** areas. **(C,D)** Confocal imaging of double immunostaining for D4R (magenta) and GS (green) indicated strong D4R-immunoreactivities in pial astrocytes (arrowheads) in the prelimbic area. **(E,F)** Confocal imaging of double immunostaining for D4R (magenta) and GS (green) indicated strong D4R-immunoreactivities in pial astrocytes (arrowheads) in the primary motor area. **(G,H)** In layer I of the primary motor area, astrocytes showed strong immunoreactivities for D4R (arrows). **(I,J)** In layer V, weak D4R-immunoreactivities were observed in protoplasmic astrocytes (arrows). **(K,L)** In layer II of the somatosensory area, D4R-immunoreactivities were observed in GS-negative cells (asterisks). **(M,N)** In layer V of the primary motor area, D4R-immunoreactivities were observed in a pyramidal cell-like large cell (asterisks) and its process (arrows). **(O–R)** In layer I of the primary motor area, strong and granular immunoreactivities for D4R were observed (arrowheads), which were in close vicinity of the GS-positive structure (green), but not overlapped. Nuclei were stained with Hoechst33342. Scale bars: 20 μm **(A,B)**; 10 μm; **(C,D,I–N)**; 5 μm **(E–H)**; 2 μm **(O–R)**.

Immunostaining for D4R revealed moderate immunoreactivity distributed throughout the cortical layers, with strong immunoreactivity observed on the pial surface of the cerebral cortex (Figures 1C, 4A,B). Double immunostaining for D4R and GS revealed that robust D4R-immunopositivity was present in pial and layer I astrocytes (Figures 4C–H), whereas GS-positive protoplasmic astrocytes in layers II and deeper exhibited low or negligible D4R-immunoreactivity (Figures 4I,J). In layer II, several GS-negative cells showed D4R-immunopositivity (Figures 4K,L). In layer V of the primary motor area, pyramidal cell-like large cells exhibited D4R-immunoreactivity in their somata and their processess (Figures 4M,N). High-magnification observations revealed that granular D4R-immunoreactivities in the neuropil of all cortical layers, which were not overlapped with GS-immunoreactivities but were in close proximity to GS-positive astrocytic structures (Figures 4O–R).

### 3.5 D5R-immunoreactivities in cortical astrocytes

**Figure 5:**
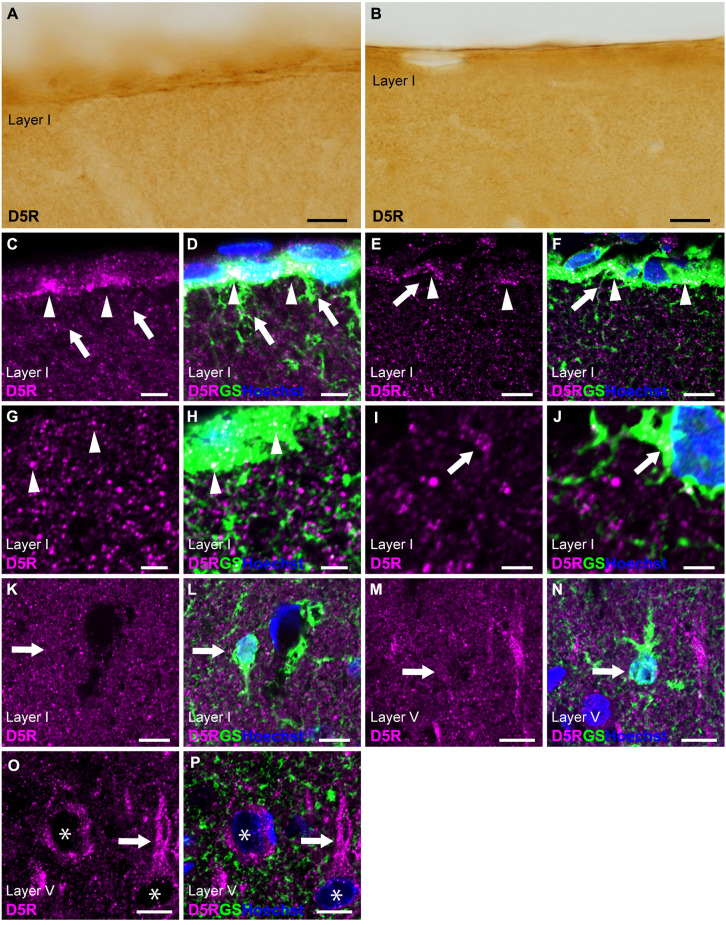
D5R-immunoreactivity in astrocytes. **(A,B)** Moderate D5R-immunoreactivities of the cortical surface of the prelimbic **(A)** and somatosensory **(B)** areas. **(C,D)** Confocal imaging of double immunostaining for D5R (magenta) and GS (green) indicated D5R-immunoreactivities biased toward the parenchymal side within pial astrocytes (arrowheads) in the prelimbic area. Processes of pial astrocytes (arrows) had only a few D5R-immunoreactivities. **(E,F)** D5R-immunoreactivities in the parenchymal side within pial astrocytes (arrowheads) in the somatosensory area. Processes of pial astrocytes (arrows) had only a few D5R-immunoreactivities. **(G,H)** In layer I of the primary motor area, astrocytes showed immunoreactivities for D5R (arrowheads). Strong and granular immunoreactivities for D5R were observed (magenta), which were in close vicinity of GS-positive structure (green), but not overlapped. **(I,J)** Confocal imaging of double immunostaining for D5R (magenta) and GS (green) indicated D5R-immunoreactivities in layer I astrocytes (arrows) in the primary motor area. **(K,L)** In layer I of the primary motor area, GS-positive astrocytes showed weak immunoreactivities for D5R (arrows). **(M,N)** In layer V of the primary motor area, GS-positive protoplasmic astrocytes (arrows) showed weak D5R-immunoreactivities. **(O,P)** In layer V of the primary motor area, strong D5R-immunoreactivities were observed in pyramidal cell-like large cells (asterisks) and their processes (arrows). Nuclei were stained with Hoechst33342. Scale bars: 20 μm **(A,B)**; 10 μm **(C–F,K–P)**; 5 μm **(G–J)**.

Similar to D4R, immunostaining for D5R revealed moderate immunoreactivity throughout the cortical layers, with moderate immunoreactivity on the pial surface of the cerebral cortex (Figures 1D, 5A,B). Double immunostaining for D5R and GS revealed that moderate D5R-positive stainings were found in pial and layer I astrocytes (Figures 5C–H). In contrast to D4R-immunoreactivity observed in the entire somata of pial astrocytes (Figures 4C–F), D5R-immunoreactivity in pial astrocytes was primarily localized to the cortical side of their somata (Figures 5C–F). Both thick and thin processes of pial astrocytes extending into layer I did not exhibit D5R-immunoreactivity (Figures 5C–F). In contrast D4R, layer I astrocytes displayed weak D5R-immunoreactivity (Figures 5I–L). GS-positive protoplasmic astrocytes in layers II and deeper displayed low or negligible D5R-immunoreactivities (Figures 5M,N). Higher magnification observation revealed granular D5R-immunoreactivities in the neuropil of all cortical layers, which were not overlapped with GS-immunoreactivities, but were in close proximity to GS-positive astrocytic structures (Figures 5G–J). In layer V of the motor cortex, pyramidal cell-like large cells exhibited D5R-immunoreactivity in their somata and their processes (Figures 5O,P).

## 4 Discussion

### 4.1 Differential dopamine receptor immunoreactivities among cortical astrocytes

**Figure 6:**
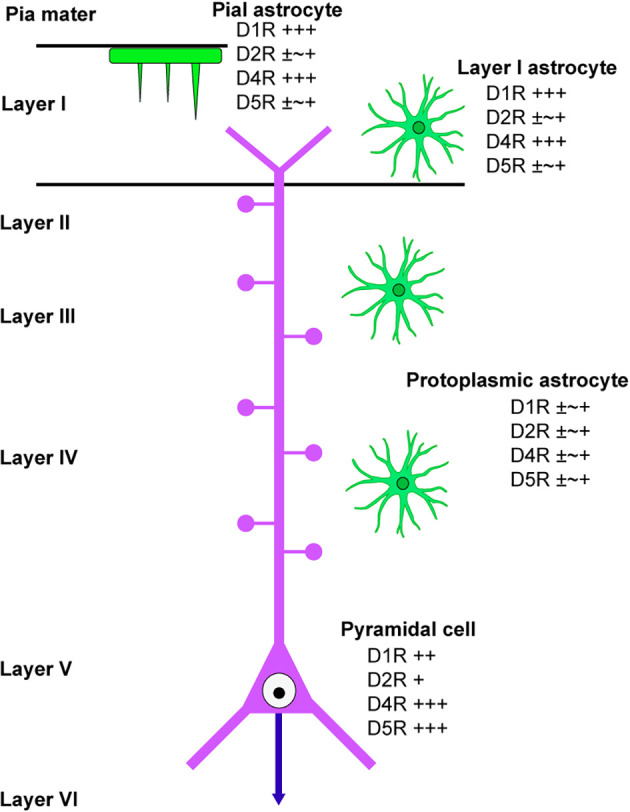
Scheme of dopamine receptor localization in cortical astrocytes. A scheme summarizes the immunolocalization of dopamine receptors in cortical astrocytes. Protoplasmic astrocytes in layer II and deeper layers are similar in terms of dopamine receptor immunolocalization, which is different from layer I astrocytes. A pyramidal cell in layer V of the primary motor area is also indicated.

This study has revealed the differential distribution of dopamine receptors in cortical astrocytes (summarized in Figure 6). Pronounced immunoreactivity for D1R and D4R was observed in the somata and thick processes of pial astrocytes. Additionally, layer I astrocytes displayed robust labeling for D1R and D4R in both their somata and processes. Conversely, protoplasmic astrocytes in layer II and deeper layers exhibited low or negligible immunoreactivities for dopamine receptors. These findings suggest that pial and layer I astrocytes are different from astrocytes in layer II and deeper layers with regard to their potential responsiveness to neuromodulators such as dopamine. Consistently, astrocytes in layer I exhibit intracellular calcium dynamics that differ from those of layer II and III astrocytes (Takata and Hirase, 2008). Given that the somata and thick processes of pial and layer I astrocytes are cholinergic M1R-immunopositive (Oda et al., 2018), it is possible that pial and layer I astrocytes possess receptors for both dopamine and acetylcholine.

### 4.2 Possible dopamine actions on pial and layer I astrocytes

In accordance with the presence of dopamine receptors in pial and layer I astrocytes, it has been observed that there is a high density of tyrosine hydroxylase (TH)-positive fibers and dopamine transport (DAT)-positive fibers in layer I, with a much lower density in layer II and deeper layer in rodents (Freed et al., 1995; Kritzer, 1998) and primates (Zubair et al., 2021). Dopaminergic fibers projecting to the cerebral cortex are derived from the ventral tegmental area (Aransay et al., 2015; Zubair et al., 2021). The distribution patterns of TH-labeled fibers and DAT-labeled fibers are highly similar. In the primate prefrontal cortex, it has been found that 92.5% of TH-labeled axons were also immunoreactive for DAT, and 97.6% of DAT-labeled axons were also immunoreactive for TH (Lewis et al., 2001). Given that dopamine release is primarily regulated through volume transmission from TH-positive fibers (Fuxe et al., 2015), it is thought that locally released dopamine can modulate the activity of pial and layer I astrocytes. Considering the conserved presence of pial astrocytes (Falcone et al., 2019) and abundant TH-positive fibers in layer I in primates (Lewis et al., 1987), dopamine receptor expression in pial astrocytes may also be conserved in other mammals.

Almost all pial astrocytes were immunolabeled with D1R and D4R. Given that Gs signaling *via* D1-type receptors and Gi singling *via* D2-type receptors, including D4R, are inversely related to cAMP production (Ledonne and Mercuri, 2017; Klein et al., 2019), the effect of dopamine on the cAMP-dependent signaling pathway in pial and layer I astrocytes may not be straightforward or unidirectional. Furthermore, it has been demonstrated that cells expressing both D1- and D2-type receptors have a greater dopamine-induced increase in intracellular Ca^2+^ concentration than cells expressing either one (Liu et al., 2009; Fischer et al., 2020). Some studies have also indicated that dopamine receptors can directly interact with other types of dopamine receptors or G protein-coupled receptors for other neuromodulators. For example, a dimer composed of D1- and D2-type receptors increases intracellular Ca^2+^ concentration (Ledonne and Mercuri, 2017; Klein et al., 2019). As previously demonstrated, almost all pial astrocytes are immunolabeled with M1R (Oda et al., 2018), in addition to D1R and D4R. An electrophysiological study reported that simultaneous stimulation of D1R and M1R leads to an enhancement of cAMP formation (Olianas et al., 2013). Further study is necessary to examine how dopamine regulates pial and layer I astrocytes.

### 4.3 Dopaminergic effects of astrocytes on pyramidal cells

Granular structures or puncta displaying robust immunoreactivities for D4R and D5R were observed in all layers of the cerebral cortex, which may be dendritic spines as we previously reported for D5R and cholinergic M1R (Oda et al., 2010, 2018). The current finding that D4R- and D5R-immunoreactive granular structures are in close proximity to GS-positive astrocytic structures is consistent with astrocytic involvement of tripartite synapses (Allen and Eroglu, 2017; Semyanov and Verkhratsky, 2021). Although astrocytic involvement of tripartite synapses is typically attributed to protoplasmic astrocytes, the presence of pial astrocytic processes extending into layer I and in close proximity to D4R- and D5R-immunoreactive granular structures suggests that pial astrocytic processes may also play a role in the regulation of synaptic transmission. Furthermore, pial and layer I astrocytes can modulate dendritic tufts of pyramidal cells, which are crucial sites for modulating the firing modes of pyramidal cells between regular firing and burst firing modes (Larkum and Zhu, 2002). These implications of this study warrant further investigation.

### 4.4 Limitations of this study

The present study has several limitations. Since the aim of this study was to investigate the localization of dopamine receptors in cortical astrocytes, we only used GS, a marker for astrocytes. Cell types other than astrocytes have not been identified with cell markers. Therefore, pyramidal cells in the primary motor cortex were used for the current figures because pyramidal cells in layer V of the primary motor cortex can be distinguished by their characteristic morphology and size.

The specificity of the primary antibodies used in this study has been verified *via* western blot analysis of tissue or cell homogenates. However, the specificity of these antibodies for immunohistochemistry has not been verified using dopamine receptor-deficient mouse brains. Given that immunostaining with each dopamine receptor antibody produces antibody-specific reactions and is consistent with the localization observed using dopamine receptor gene-modified mice, such as D1R-tdTomato mice (Anastasiades et al., 2019), D2R-EGFP mice (Khlghatyan et al., 2019), and D4R-EGFP mice (Noaín et al., 2006), the current results may reflect genuine localization of dopamine receptors in the cerebral cortex. In general, the visualization of G protein-coupled receptors is not easy for immunostaining and *in situ* hybridization. Recently developed modified *in situ* hybridization chain reaction may work to detect dopamine receptors in cortical neurons and glia in a quantitative manner (Tsuneoka and Funato, 2020).

## Data availability statement

The original contributions presented in the study are included in the article, further inquiries can be directed to the corresponding author/s.

## Ethics statement

The animal study was reviewed and approved by Institutional Animal Care and Use Committee of Toho University.

## Author contributions

SO and HF had full access to all the data in the study and take responsibility for the integrity of the data and the accuracy of data analysis. SO and HF: study concept and design, analysis and interpretation of data, manuscript writing. All authors contributed to the article and approved the submitted version.

## References

[B1] Aira Z. Barrenetxea T. Buesa I. García Del Caño G. Azkue J. J. (2016). Dopamine D1-like receptors regulate constitutive, μ-opioid receptor-mediated repression of use-dependent synaptic plasticity in dorsal horn neurons: more harm than good? J. Neurosci. 36, 5661–5673. 10.1523/JNEUROSCI.2469-15.2016 27194343 PMC6601769

[B2] Allen N. J. Eroglu C. (2017). Cell biology of astrocyte-synapse interactions. Neuron 96, 697–708. 10.1016/j.neuron.2017.09.056 29096081 PMC5687890

[B3] Anastasiades P. G. Boada C. Carter A. G. (2019). Cell-type-specific D1 dopamine receptor modulation of projection neurons and interneurons in the prefrontal cortex. Cereb. Cortex 29, 3224–3242. 10.1093/cercor/bhy299 30566584 PMC6611468

[B4] Andriezen W. L. (1893). The neuroglia elements in the human brain. Br. Med. J. 2, 227–230. 10.1136/bmj.2.1700.227 20754383 PMC2422013

[B5] Aransay A. Rodríguez-López C. García-Amado M. Clascá F. Prensa L. (2015). Long-range projection neurons of the mouse ventral tegmental area: a single-cell axon tracing analysis. Front. Neuroanat. 9:59. 10.3389/fnana.2015.00059 26042000 PMC4436899

[B6] Batiuk M. Y. Martirosyan A. Wahis J. de Vin F. Marneffe C. Kusserow C.. (2020). Identification of region-specific astrocyte subtypes at single cell resolution. Nat. Commun. 11:1220. 10.1038/s41467-019-14198-8 32139688 PMC7058027

[B7] Colombo J. A. (2017). The interlaminar glia: from serendipity to hypothesis. Brain Struct. Funct. 222, 1109–1129. 10.1007/s00429-016-1332-8 27864630

[B8] Colombo J. A. Yáñez A. Puissant V. Lipina S. (1995). Long, interlaminar astroglial cell processes in the cortex of adult monkeys. J. Neurosci. Res. 40, 551–556. 10.1002/jnr.490400414 7616615

[B9] Diaz J. Lévesque D. Lammers C. H. Griffon N. Martres M. P. Schwartz J. C.. (1995). Phenotypical characterization of neurons expressing the dopamine D3 receptor in the rat brain. Neuroscience 65, 731–745. 10.1016/0306-4522(94)00527-c 7609872

[B10] Falcone C. Wolf-Ochoa M. Amina S. Hong T. Vakilzadeh G. Hopkins W. D.. (2019). Cortical interlaminar astrocytes across the therian mammal radiation. J. Comp. Neurol. 527, 1654–1674. 10.1002/cne.24605 30552685 PMC6465161

[B11] Fischer T. Scheffler P. Lohr C. (2020). Dopamine-induced calcium signaling in olfactory bulb astrocytes. Sci. Rep. 10:631. 10.1038/s41598-020-57462-4 31959788 PMC6971274

[B12] Freed C. Revay R. Vaughan R. A. Kriek E. Grant S. Uhl G. R.. (1995). Dopamine transporter immunoreactivity in rat brain. J. Comp. Neurol. 359, 340–349. 10.1002/cne.903590211 7499533

[B13] Fuxe K. Agnati L. F. Marcoli M. Borroto-Escuela D. O. (2015). Volume transmission in central dopamine and noradrenaline neurons and its astroglial targets. Neurochem. Res. 40, 2600–2614. 10.1007/s11064-015-1574-5 25894681

[B14] García-Marqués J. López-Mascaraque L. (2013). Clonal identity determines astrocyte cortical heterogeneity. Cereb. Cortex 23, 1463–1472. 10.1093/cercor/bhs134 22617854

[B15] Hazelwood L. A. Free R. B. Cabrera D. M. Skinbjerg M. Sibley D. R. (2008). Reciprocal modulation of function between the D1 and D2 dopamine receptors and the Na^+^,K^+^-ATPase. J. Biol. Chem. 283, 36441–36453. 10.1074/jbc.M805520200 18984584 PMC2605984

[B16] Howe M. W. Feig S. L. Osting S. M. K. Haberly L. B. (2008). Cellular and subcellular localization of Kir2.1 subunits in neurons and glia in piriform cortex with implications for K^+^ spatial buffering. J. Comp. Neurol. 506, 877–893. 10.1002/cne.21534 18076085

[B17] Khlghatyan J. Quintana C. Parent M. Beaulieu J.-M. (2019). High sensitivity mapping of cortical dopamine D2 receptor expressing neurons. Cereb. Cortex 29, 3813–3827. 10.1093/cercor/bhy261 30295716 PMC6686758

[B18] Klein M. O. Battagello D. S. Cardoso A. R. Hauser D. N. Bittencourt J. C. Correa R. G. (2019). Dopamine: functions, signaling and association with Neurological diseases. Cell. Mol. Neurobiol. 39, 31–59. 10.1007/s10571-018-0632-3 30446950 PMC11469830

[B19] Kritzer M. F. (1998). Perinatal gonadectomy exerts regionally selective, lateralized effects on the density of axons immunoreactive for tyrosine hydroxylase in the cerebral cortex of adult male rats. J. Neurosci. 18, 10735–10748. 10.1523/JNEUROSCI.18-24-10735.1998 9852608 PMC6793338

[B20] Kulijewicz-Nawrot M. Syková E. Chvátal A. Verkhratsky A. Rodríguez J. J. (2013). Astrocytes and glutamate homoeostasis in Alzheimer’s disease: a decrease in glutamine synthetase, but not in glutamate transporter-1, in the prefrontal cortex. ASN Neuro 5, 273–282. 10.1042/AN20130017 24059854 PMC3791522

[B21] Lanjakornsiripan D. Pior B.-J. Kawaguchi D. Furutachi S. Tahara T. Katsuyama Y.. (2018). Layer-specific morphological and molecular differences in neocortical astrocytes and their dependence on neuronal layers. Nat. Commun. 9:1623. 10.1038/s41467-018-03940-3 29691400 PMC5915416

[B22] Larkum M. E. Zhu J. J. (2002). Signaling of layer 1 and whisker-evoked Ca^2+^ and Na^+^ action potentials in distal and terminal dendrites of rat neocortical pyramidal neurons *in vivo* and *in vivo*. J. Neurosci. 22, 6991–7005. 10.1523/JNEUROSCI.22-16-06991.2002 12177197 PMC6757874

[B23] Le Moine C. Bloch B. (1996). Expression of the D3 dopamine receptor in peptidergic neurons of the nucleus accumbens: comparison with the D1 and D2 dopamine receptors. Neuroscience 73, 131–143. 10.1016/0306-4522(96)00029-2 8783237

[B24] Ledonne A. Mercuri N. B. (2017). Current concepts on the physiopathological relevance of dopaminergic receptors. Front. Cell. Neurosci. 11:27. 10.3389/fncel.2017.00027 28228718 PMC5296367

[B25] Lee S. P. So C. H. Rashid A. J. Varghese G. Cheng R. Lança A. J.. (2004). Dopamine D1 and D2 receptor co-activation generates a novel phospholipase C-mediated calcium signal. J. Biol. Chem. 279, 35671–35678. 10.1074/jbc.M401923200 15159403

[B26] Lewis D. A. Campbell M. J. Foote S. L. Goldstein M. Morrison J. H. (1987). The distribution of tyrosine hydroxylase-immunoreactive fibers in primate neocortex is widespread but regionally specific. J. Neurosci. 7, 279–290. 10.1523/JNEUROSCI.07-01-00279.1987 2879896 PMC6568855

[B27] Lewis D. A. Melchitzky D. S. Sesack S. R. Whitehead R. E. Auh S. Sampson A. (2001). Dopamine transporter immunoreactivity in monkey cerebral cortex: regional, laminar and ultrastructural localization. J. Comp. Neurol. 432, 119–136. 10.1002/cne.1092 11241381

[B28] Liu J. Wang F. Huang C. Long L.-H. Wu W.-N. Cai F.. (2009). Activation of phosphatidylinositol-linked novel D1 dopamine receptor contributes to the calcium mobilization in cultured rat prefrontal cortical astrocytes. Cell. Mol. Neurobiol. 29, 317–328. 10.1007/s10571-008-9323-9 18975071 PMC11505845

[B29] Liu X. Zhang Z. Guo W. Burnstock G. He C. Xiang Z. (2013). The superficial glia limitans of mouse and monkey brain and spinal cord. Anat. Rec. 296, 995–1007. 10.1002/ar.22717 23674345

[B30] Luedtke R. R. Griffin S. A. Conroy S. S. Jin X. Pinto A. Sesack S. R. (1999). Immunoblot and immunohistochemical comparison of murine monoclonal antibodies specific for the rat D1a and D1b dopamine receptor subtypes. J. Neuroimmunol. 101, 170–187. 10.1016/s0165-5728(99)00142-3 10580800

[B31] Mladinov M. Mayer D. Brčić L. Wolstencroft E. Man N. Holt I.. (2010). Astrocyte expression of D2-like dopamine receptors in the prefrontal cortex. Transl. Neurosci. 1, 238–243. 10.2478/v10134-010-0035-6

[B32] Mrzljak L. Bergson C. Pappy M. Huff R. Levenson R. Goldman-Rakic P. S. (1996). Localization of dopamine D4 receptors in GABAergic neurons of the primate brain. Nature 381, 245–248. 10.1038/381245a0 8622768

[B33] Nasonkin I. O. Lazo K. Hambright D. Brooks M. Fariss R. Swaroop A. (2011). Distinct nuclear localization patterns of DNA methyltransferases in developing and mature mammalian retina. J. Comp. Neurol. 519, 1914–1930. 10.1002/cne.22613 21452232 PMC12058000

[B34] Noaín D. Avale M. E. Wedemeyer C. Calvo D. Peper M. Rubinstein M. (2006). Identification of brain neurons expressing the dopamine D4 receptor gene using BAC transgenic mice. Eur. J. Neurosci. 24, 2429–2438. 10.1111/j.1460-9568.2006.05148.x 17100831

[B35] Norenberg M. D. Martinez-Hernandez A. (1979). Fine structural localization of glutamine synthetase in astrocytes of rat brain. Brain Res. 161, 303–310. 10.1016/0006-8993(79)90071-4 31966

[B36] Oda S. Funato H. Adachi-Akahane S. Ito M. Okada A. Igarashi H.. (2010). Dopamine D5 receptor immunoreactivity is differentially distributed in GABAergic interneurons and pyramidal cells in the rat medial prefrontal cortex. Brain Res. 1329, 89–102. 10.1016/j.brainres.2010.03.011 20226768

[B37] Oda S. Tsuneoka Y. Yoshida S. Adachi-Akahane S. Ito M. Kuroda M.. (2018). Immunolocalization of muscarinic M1 receptor in the rat medial prefrontal cortex. J. Comp. Neurol. 526, 1329–1350. 10.1002/cne.24409 29424434 PMC5900831

[B38] Olianas M. C. Dedoni S. Onali P. (2013). Coincidence signaling of dopamine D1-like and M1 muscarinic receptors in the regulation of cyclic AMP formation and CREB phosphorylation in mouse prefrontal cortex. Neurosignals 21, 61–74. 10.1159/000335208 22456324

[B39] Ricci A. Bronzetti E. Mannino F. Mignini F. Morosetti C. Tayebati S. K.. (2001). Dopamine receptors in human platelets. Naunyn Schmiedebergs Arch. Pharmacol. 363, 376–382. 10.1007/s002100000339 11330330

[B40] Ricci A. Marchal-Victorion S. Bronzetti E. Parini A. Amenta F. Tayebati S. K. (2002). Dopamine D4 receptor expression in rat kidney: evidence for pre- and postjunctional localization. J. Histochem. Cytochem. 50, 1091–1096. 10.1177/002215540205000811 12133912

[B41] Robinson S. R. (2001). Changes in the cellular distribution of glutamine synthetase in Alzheimer’s disease. J. Neurosci. Res. 66, 972–980. 10.1002/jnr.10057 11746426

[B42] Rua R. McGavern D. B. (2018). Advances in meningeal immunity. Trends Mol. Med. 24, 542–559. 10.1016/j.molmed.2018.04.003 29731353 PMC6044730

[B43] Semyanov A. Verkhratsky A. (2021). Astrocytic processes: from tripartite synapses to the active milieu. Trends Neurosci. 44, 781–792. 10.1016/j.tins.2021.07.006 34479758

[B44] Sofroniew M. V. (2020). Astrocyte reactivity: subtypes, states and functions in CNS innate immunity. Trends Immunol. 41, 758–770. 10.1016/j.it.2020.07.004 32819810 PMC7484257

[B45] Tabata H. (2015). Diverse subtypes of astrocytes and their development during corticogenesis. Front. Neurosci. 9:114. 10.3389/fnins.2015.00114 25904839 PMC4387540

[B46] Takata N. Hirase H. (2008). Cortical layer 1 and layer 2/3 astrocytes exhibit distinct calcium dynamics *in vivo*. PLoS One 3:e2525. 10.1371/journal.pone.0002525 18575586 PMC2424136

[B47] Tsuneoka Y. Funato H. (2020). Modified *in situ* hybridization chain reaction using short hairpin DNAs. Front. Mol. Neurosci. 13:75. 10.3389/fnmol.2020.00075 32477063 PMC7235299

[B48] Verkhratsky A. Nedergaard M. (2018). Physiology of astroglia. Physiol. Rev. 98, 239–389. 10.1152/physrev.00042.2016 29351512 PMC6050349

[B49] Zeisel A. Hochgerner H. Lönnerberg P. Johnsson A. Memic F. van der Zwan J.. (2018). Molecular architecture of the mouse nervous system. Cell 174, 999–1014.e22. 10.1016/j.cell.2018.06.021 30096314 PMC6086934

[B50] Zeisel A. Muñoz-Manchado A. B. Codeluppi S. Lönnerberg P. La Manno G. Juréus A.. (2015). Brain structure. cell types in the mouse cortex and hippocampus revealed by single-cell RNA-seq. Science 347, 1138–1142. 10.1126/science.aaa1934 25700174

[B51] Zubair M. Murris S. R. Isa K. Onoe H. Koshimizu Y. Kobayashi K.. (2021). Divergent whole brain projections from the ventral midbrain in macaques. Cereb. Cortex 31, 2913–2931. 10.1093/cercor/bhaa399 33558867 PMC8107798

